# Aurora A–mediated pyruvate kinase M2 phosphorylation promotes biosynthesis with glycolytic metabolites and tumor cell cycle progression

**DOI:** 10.1016/j.jbc.2022.102561

**Published:** 2022-10-02

**Authors:** Ya Jiang, Ting Wang, Dandan Sheng, Chaoqiang Han, Tian Xu, Peng Zhang, Weiyi You, Weiwei Fan, Zhiyong Zhang, Tengchuan Jin, Xiaotao Duan, Xiao Yuan, Xing Liu, Kaiguang Zhang, Ke Ruan, Jue Shi, Jing Guo, Aoxing Cheng, Zhenye Yang

**Affiliations:** 1 Department of Digestive Disease, The First Affiliated Hospital of University of Science and Technology of China, Anhui Provincial Hospital, Hefei, China; 2 MOE Key Laboratory for Cellular Dynamics, University of Science and Technology of China, Hefei, China; 3 State Key Laboratory of Toxicology and Medical Countermeasures, Beijing Institute of Pharmacology and Toxicology, Beijing, China; 4 Anhui Key Laboratory of Cellular Dynamics and Chemical Biology &CAS Center of Excellence in Molecular Cell Sciences, Hefei, Anhui, China; 5 Department of Physics and Department of Biology, Center for Quantitative Systems Biology, Hong Kong Baptist University, Hong Kong, China; 6 Biomedical Sciences and Health Laboratory of Anhui Province, University of Science & Technology of China, Hefei, China

**Keywords:** Aurora A, PKM2, phosphorylation, biosynthesis, cell cycle progression, 2PG, phosphoglycerate, co-IP, coimmunoprecipitation, G6P, glucose 6-phosphate, KD, kinase-dead, MS, mass spectrometry, PEP, phosphoenolpyruvate, PLA, proximal ligation assay, SEC, size-exclusion chromatography

## Abstract

Cancer cells have distinctive demands for intermediates from glucose metabolism for biosynthesis and energy in different cell cycle phases. However, how cell cycle regulators and glycolytic enzymes coordinate to orchestrate the essential metabolic processes are still poorly characterized. Here, we report a novel interaction between the mitotic kinase, Aurora A, and the glycolytic enzyme, pyruvate kinase M2 (PKM2), in the interphase of the cell cycle. We found Aurora A–mediated phosphorylation of PKM2 at threonine 45. This phosphorylation significantly attenuated PKM2 enzymatic activity by reducing its tetramerization and also promoted glycolytic flux and the branching anabolic pathways. Replacing the endogenous PKM2 with a nonphosphorylated PKM2 T45A mutant inhibited glycolysis, glycolytic branching pathways, and tumor growth in both *in vitro* and *in vivo* models. Together, our study revealed a new protumor function of Aurora A through modulating a rate-limiting glycolytic enzyme, PKM2, mainly during the S phase of the cell cycle. Our findings also showed that although both Aurora A and Aurora B kinase phosphorylate PKM2 at the same residue, the spatial and temporal regulations of the specific kinase and PKM2 interaction are context dependent, indicating intricate interconnectivity between cell cycle and glycolytic regulators.

Aerobic glycolysis is a common hallmark of cancer cells, and accumulation of glycolytic metabolites for endogenous biosynthesis is known to facilitate tumor growth (1, 2, 3, 4). As tumor cells have variable demands for building blocks and energy in distinctive cell cycle phases, coordination of cell cycle regulators and metabolic enzymes are crucial for an efficient cell cycle progression that underlies tumor proliferation (5, 6, 7). Indeed, glucose metabolism has been reported to be modulated by key cell cycle regulators. For instance, PFKFB3 (6-phosphofructo-2-kinase/fructose-2, 6-biphosphatase 3) is degraded after mitotic exit in an APC/C (anaphase-promoting complex/cyclosome)-Cdh1–dependent manner until mid-to-late G1 phase, when Cdh1 is inactivated by interphase Cdks (cyclin-dependent kinases) (8). On the other hand, PFKFB3 can promote entry of mitosis by increasing the expression of Cdk1 and Cdc25 (9). PFKFB3 can also translocate to the nucleus to activate or inactivate cell cycle regulators. F-2,6-BP (fructose 2,6-bisphosphate), a product of nuclear PFKFB3, was found to represses cell cycle inhibitor p27kip1 to promote G1/S transition (8). Cdk2 phosphorylates glycolytic enzyme triosephosphate isomerase 1 (TPI1) to promote its nuclear translocation and histone acetylation for cell cycle progression (10). Another example of interdependence of cell cycle regulators and glycolytic enzymes is that cyclin D3/Cdk6 promotes the oxidative branch of the pentose phosphate pathway and serine pathways by inhibiting PFK1 (phosphofructokinase-1) and PKM2 (pyruvate kinase M2) (11).

PKM2, a rate-limiting enzyme in glycolysis, functions in a number of pathways in the nucleus in different cell cycle stages (12, 13). Tetrameric PKM2 efficiently transfers a phosphate group from phosphoenolpyruvate (PEP) to ADP and subsequently generates pyruvate and ATP, while dimeric PKM2 has much lower enzymatic activity and limits the pyruvate production at the end of glycolysis, redirecting the glycolytic metabolites toward biosynthesis pathways (13, 14). In the G1 phase, monomeric PKM2 can translocate to the nucleus to increase cyclin D1 expression by regulating c-*myc* and β-catenin. c-*myc* in turn can transcriptionally upregulate glycolytic enzymes LDHA, GLUT1, and PKM2 (15). This function of PKM2 in the nucleus can be promoted by Cdc25A dephosphorylation (16). PKM2 also functions as a protein kinase to phosphorylate histone (17), Bub3 (18), MLC2 (19) and promotes interphase progression, chromosome segregation, and cytokinesis. Overall, previous studies illustrate PKM2 is tightly interconnected with cell cycle regulation through reciprocal activation (5).

Aurora A, a conserved serine/threonine kinase, is involved in centrosome maturation in G2 phase and bipolar spindle formation in mitosis (20, 21). Aurora A is found to be overexpressed in various solid tumors (22, 23), and overexpression of Aurora A kinase plays an important role in metabolic reprogramming-mediated cancer progression (24, 25). In our previous study, we found that Aurora A directly binds and phosphorylates the glycolytic enzyme LDHB at S162, which increases its activity in reducing pyruvate to lactate and efficiently promotes NAD^+^ regeneration and glycolysis (25). Based on the proteomic analysis results, we also identified PKM2 as another glycolytic enzyme that strongly interacts with Aurora A (25). Given the crucial role of PKM2 in the glycolysis of cancer cells, in this study, we sought to elucidate the functional regulation of PMK2 by Aurora A kinase and its impact on tumor growth.

## Results

### Aurora A directly binds PKM2 both *in vitro* and *in vivo*

**Figure 1:**
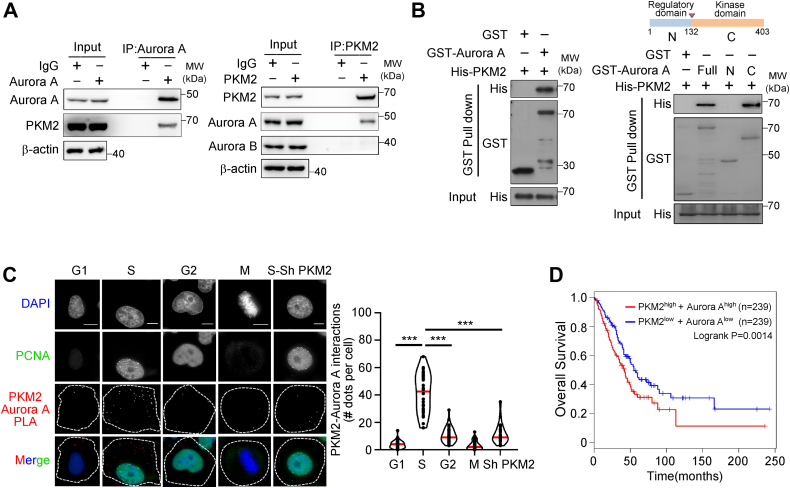
**Aurora A directly binds PKM2 both*in vitro* and *in vivo*.***A*, co-IP was conducted with Aurora A (left panel) or PKM2 (right panel) antibody in H1299 cell extracts. The elutes were subjected to immunoblotting with the indicated antibodies. *B*, GST pull-down assay was performed with GST-Aurora A (left panel) or GST-Aurora A truncations (right panel) with His-PKM2. The elutes were subjected to immunoblotting with the indicated antibodies. *C*, proximity ligation assay (PLA) was performed in H1299 cells with Aurora A and PKM2 antibodies. DAPI and PCNA were cell cycle indicators. The cell cycle–dependent interaction between Aurora A and PKM2 was shown (left panel). The scale bar represents 10 μm. The PLA signals in interphase and mitotic cell were quantified (right panel) (G1, n = 59; S, n = 34; G2, n = 44; M, n = 53; Sh PKM, n = 21). *D*, overall survival was compared between samples with PKM2^high^ Aurora-A^high^ and PKM2^low^ Aurora-A^low^ expression classified by median expression level in the lung adenocarcinoma, analyzed by the GEPIA2 bioinformatics website. DAPI, 4′,6-diamidino-2-phenylindole.

To confirm whether Aurora A interacts with PKM2, coimmunoprecipitation (co-IP) and glutathione-*S*-transferase (GST) pull-down assay were conducted. The reciprocal co-IP using both Aurora A and PKM2 antibodies showed that endogenous Aurora A bound PKM2 in H1299 cancer cells (Fig. 1*A*). In contrast, in this lung cancer cell line PKM2 did not bind Aurora B, the other member of Aurora kinase family, despite both kinases share high homology in kinase domain and common substrates (Fig. 1*A*). Moreover, *in vitro* binding assay demonstrated that GST-Aurora A could directly interact with His-tagged PKM2, and the C-terminal kinase domain of Aurora A mediated their interaction (Fig. 1*B*). As the localization and activity of Aurora A kinase changes along with the cell cycle progression, we further examined Aurora A and PKM2 interaction in different cell cycle phases by proximal ligation assay (PLA). The PLA signal confirmed the direct interaction between Aurora A and PKM2 in the cytoplasm, which occurred mainly during S phase of interphase but not in mitosis (Fig. 1*C*). Knocking down PKM2 significantly decreased the PLA dot, further confirming that the direct interaction between Aurora A and PKM2 (Fig. 1*C*).

Next, we analyzed the clinical relevance of the expression levels of Aurora A and PKM2 in cancers from published databases. The clinical data showed worse survival statistics for lung cancer patients with higher expression of both PKM2 and Aurora A (Fig. 1*D*). These results suggest interaction of Aurora A and PKM2 is particularly prevalent in lung cancer cells and high level of both Aurora A and PKM2 are associated with worse prognosis for lung cancer (13, 26).

### Aurora A phosphorylates PKM2 at threonine 45

**Figure 2:**
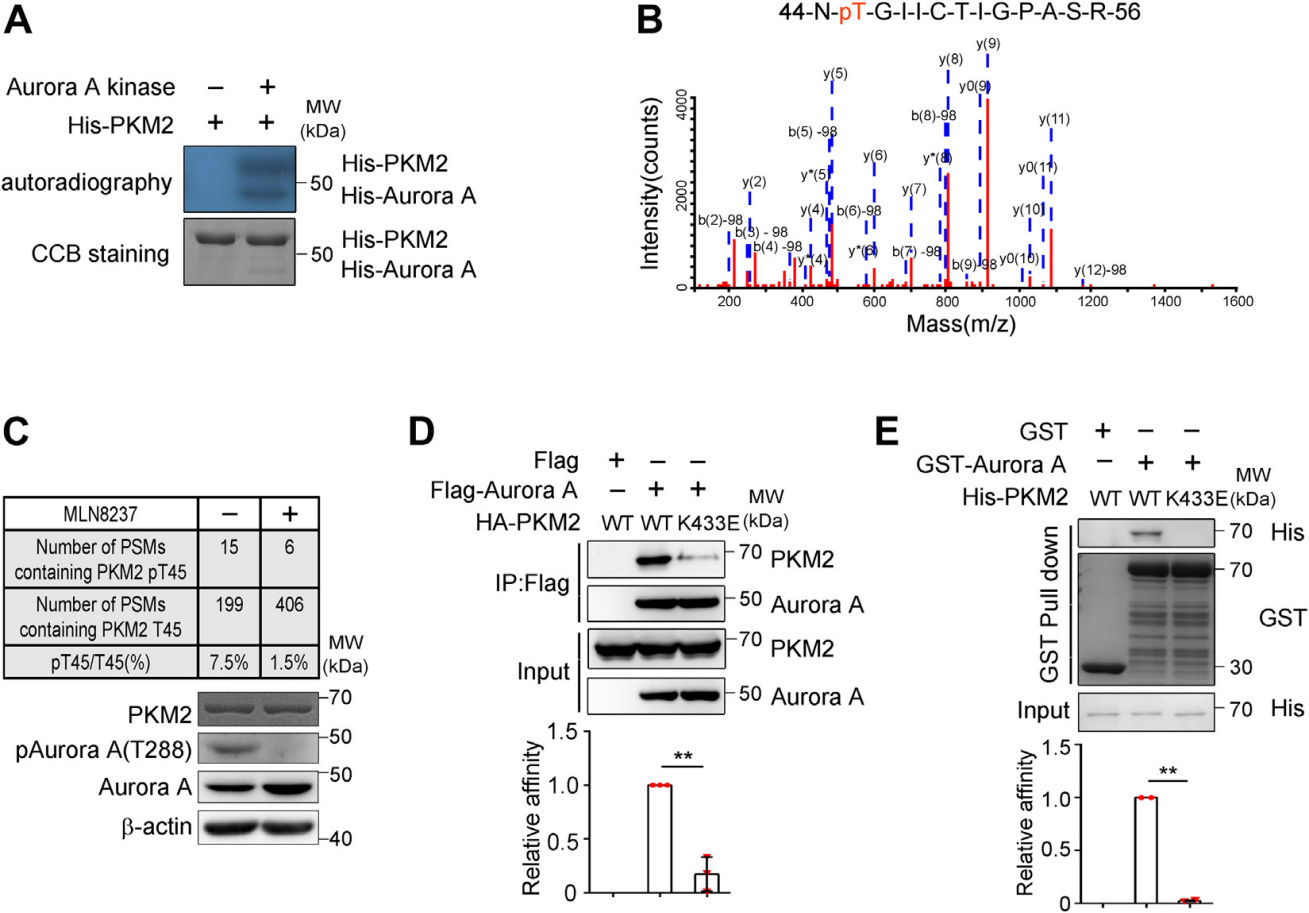
**Aurora A phosphorylates PKM2 at threonine 45.***A*, recombinant His-Aurora A and His-PKM2 were incubated with ^32^P-labeled ATP followed by SDS-PAGE. The gel was subjected to autoradiography and Coomassie blue staining. *B*, mass spectrometry (MS) data showed threonine 45 of PKM2 was phosphorylated. *C*, H1299 cells were treated with or without Aurora A kinase inhibitor, MLN8237 (100 nM). PKM2 was isolated and subjected to MS analyses. The number of PSMs (the peptide-spectrum matches) containing PKM2 T45 or PKM2 pT45 were counted (upper panel). The extracts of these cells were immunoblotted with the indicated antibodies (lower panel). *D*, Flag-Aurora A was coexpressed with HA-PKM2 or HA-PKM2 K433E mutant in 293T cells. Co-IP was conducted with Flag beads. The elutes were subjected to immunoblotting with the indicated antibodies (upper panel). Quantified data were shown (lower panel) (n = 3). *E*, GST pull-down assay was performed with GST-Aurora A and His-PKM2 or His-PKM2 K433E mutant. The elutes were subjected to immunoblotting with the indicated antibodies (upper panel). Quantified data were shown (lower panel) (n = 2). Co-IP, coimmunoprecipitation.

To elucidate the regulation underlying Aurora A and PKM2 interaction, we first tested whether PKM2 is a substrate of Aurora A kinase. *In vitro* kinase assays illustrated that Aurora A could phosphorylate PKM2 (Fig. 2*A*). Mass spectrometry (MS) analysis of purified PKM2 from *in vitro* assays (Fig. 2*B*) and in cultured H1299 cells (Fig. 2*C*) further revealed Aurora A phosphorylated PKM2 at Threonine 45 (T45), an evolutionarily conserved residue (Fig. S1*A*) that was also phosphorylated by Aurora B kinase during cytokinesis in glioblastoma cells (19). Inhibition of Aurora A with a selective inhibitor, MLN8237 (Fig. 2*C*), significantly reduced the phosphorylation of PKM2 T45 in H1299 cells, further confirming this modification *in vivo* (Fig. 2*C*). When kinase dead Aurora A was incubated with PKM2 in the *in vitro* kinase assay, the ratio of phosphorylated PKM2 T45 was also significantly reduced (Fig. S1*B*). Lysine 433 of PKM2 (K433) was previously reported to be critical for its interaction with Aurora B (19), and K433 is encoded by exon 10, an exon that is present in PKM2, but not in PKM1, after alternative splicing. We found that when K433 was mutated, the associations of PKM2 with Aurora A were also abolished in co-IP and GST pull-down assays (Fig. 2, *D* and *E*). Therefore, our data suggested both Aurora A and Aurora B bind and phosphorylate PKM2 in a similar manner, although these two kinases may have variable activities on PKM2 in different cancer cells.

### Aurora A–mediated phosphorylation of PKM2 T45 inhibited the enzymatic activity of PKM2 by reducing its tetramerization

**Figure 3:**
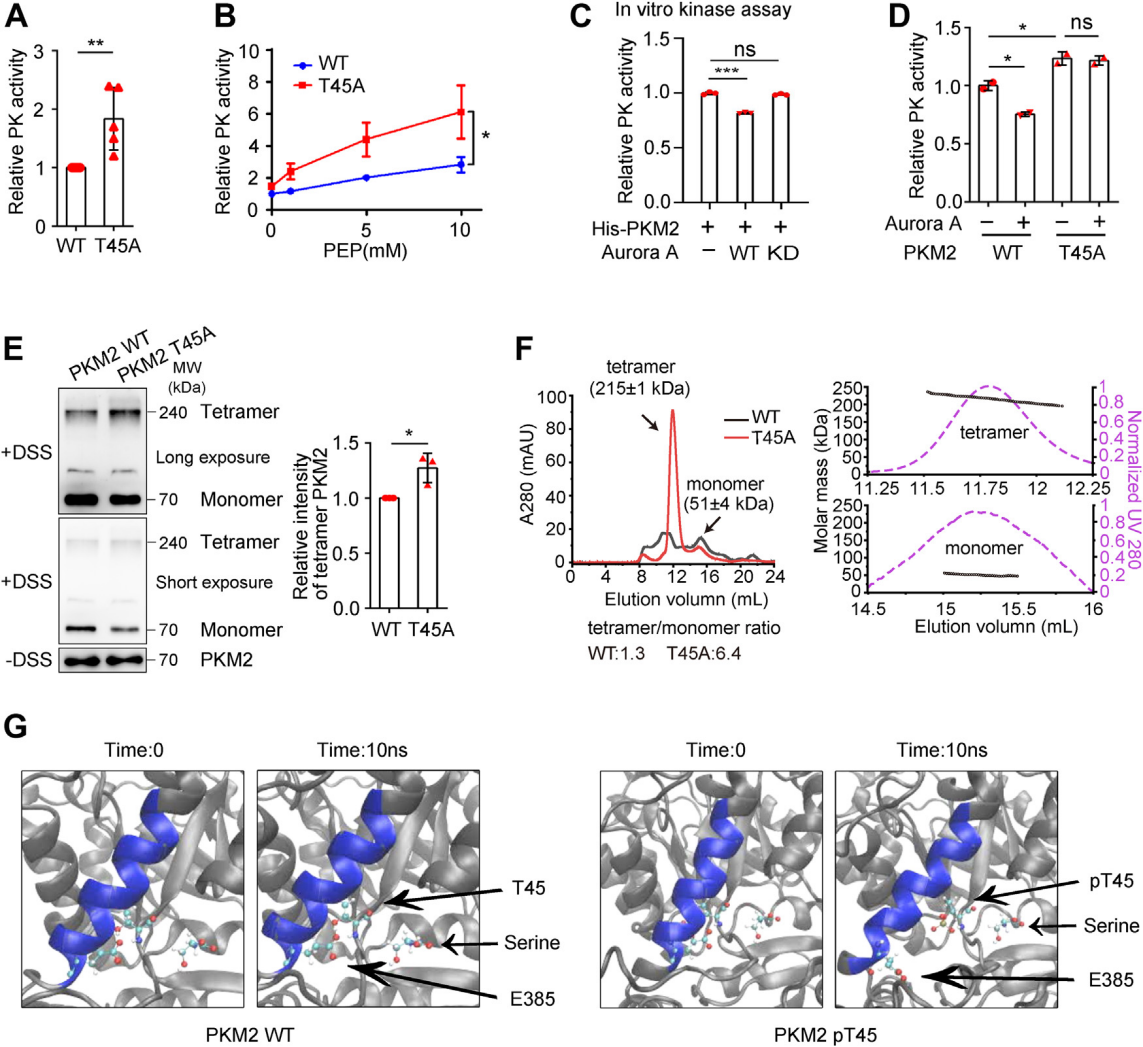
**Aurora A–mediated phosphorylation of PKM2 at T45 inhibits the enzymatic activity of PKM2 by reducing its tetramerization.***A*, His-PKM2 WT and His-PKM2 T45A mutant were expressed in *E. coli*. The purified proteins were subjected to measurement of PK activities (n = 5). *B*, the purified proteins used in (*A*) were subjected to measurement of the binding affinity toward PEP (n = 3). *C*, His-PKM2 was incubated with ATP, TPX2 (1–25 aa) and GST, GST-Aurora A, or GST-KD-Aurora A (kinase dead Aurora A, D274A mutant). The activities of PK were measured (n = 3). *D*, Aurora A was overexpressed in H1299 cells that had endogenous PKM2 knocked down and expressed shRNA-resistant Flag-PKM2 WT or Flag-PKM2 T45A. Flag-tagged proteins were purified by IP and subjected to measurement of the PK activities (n = 2). *E*, extracts of H1299 cells that had endogenous PKM2 knocked down and expressed shRNA-resistant Flag-PKM2 WT or Flag-PKM2 T45A were incubated with Flag beads. Flag-tagged proteins were purified, subjected to DSS crosslinking, and immunoblotted with the indicated antibodies (left panel). Quantified data were shown (right panel) (n = 3). *F*, size-exclusion chromatography coupled with static laser light scattering (SEC-MALS) revealed the tetramer/monomer ratio of PKM2 WT and PKM2 T45A mutant. *G*, molecular dynamics simulation revealed the conformational changes in PKM2 T45 or pT45 and E385 in 10 ns. The first and the last conformations were shown. DSS, disuccinimidyl suberate; IP, immunoprecipitation; MALS, multiangle light scattering; PEP, phosphoenolpyruvate.

Next, we sought to examine whether Aurora A–mediated phosphorylation of PKM2 at T45 modulates the catalytic activity of PKM2 in glycolysis. When T45 of PKM2 was mutated to alanine (T45A), the enzymatic activity of the nonphosphorylated mutant was significantly increased compared to that of the WT PKM2 (Fig. 3*A*). In particular, at the same substrate concentrations of PEP, the catalytic activities of PKM2 T45A mutant were much higher than those of the WT PKM2, indicating that T45A mutant had stronger binding affinity to PEP (Fig. 3*B*). In line with results from the nonphosphorylated T45A mutant, the enzymatic activity decreased when PKM2 was incubated with Aurora A, but not with the kinase dead Aurora A, *in vitro* (Fig. 3*C*).

To further examine whether the enzymatic activity of PKM2 in cancer cells was regulated by Aurora A, we generated H1299 cells that had endogenous WT PKM2 depleted and expressed either shRNA-resistant Flag-PKM2 WT or Flag-PKM2 T45A. Overexpressing Aurora A in the cancer cells inhibited the activity of PKM2 but not the PKM2 T45A mutant (Fig. 3*D*). Moreover, Aurora A inhibition with MLN8237 increased the enzymatic activity of PKM2 (Fig. S1*C*). These results indicated that Aurora A–mediated phosphorylation of PKM2 at T45 inhibited the enzymatic activity of PKM2.

To dissect the molecular mechanism underlying the attenuation of PKM2 enzymatic activity upon Aurora A–mediated phosphorylation, we examined the composition ratio of PKM2 monomer and tetramer. Disuccinimidyl suberate (DSS) crosslinking assay showed increased formation of PKM2 tetramer, the allosteric state of higher enzymatic activity, for the T45A mutant (Fig. 3*E*). In addition, size-exclusion chromatography (SEC) coupled with static laser light scattering data confirmed that the ratio of tetramer is much higher for T45A mutant than for WT PKM2 (Fig. 3*F*). These results were consistent with the enhanced activity of T45A mutant from the *in vitro* assay (Fig. 3*A*). To seek insight into the structural changes of PKM2 induced by T45 phosphorylation that may inhibit tetramer formation, we simulated the dynamic conformation using the structure of PKM2 (Protein Data Bank code: 4B2D). Molecular modeling revealed that phosphorylation of PKM2 at T45 reduced the stability of an α-helix, which is critical for the formation of tetramer, through charge repulsion with E385 (Fig. 3*G*). Together, these results suggested that Aurora A–mediated phosphorylation of PKM2 at T45 inhibits the enzyme activity of PKM2 by reducing its tetramerization.

### Phosphorylation of PKM2 at T45 promotes glycolysis and serine synthesis

**Figure 4:**
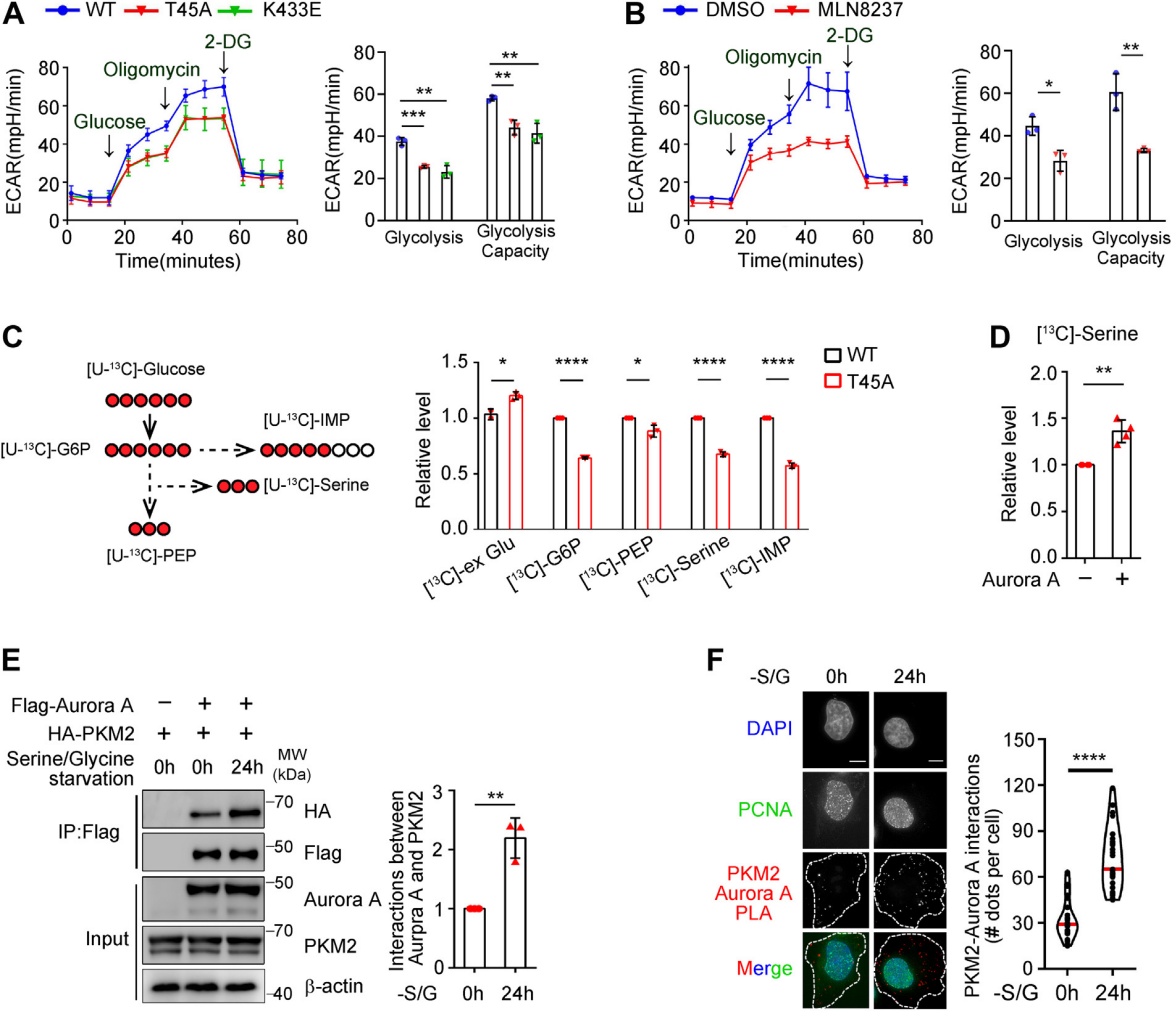
**Phosphorylation of PKM2 at T45 promotes glycolysis and serine synthesis pathway.***A*, endogenous PKM2 was knocked down in H1299 cells and shRNA resistant Flag-PKM2 WT, Flag-PKM2 T45A, or Flag-PKM2 K433E mutant were expressed. Seahorse assays were performed to evaluate the glycolytic flux. ECAR over time (left panel) and ECAR in different stages of the measurement (right panel) were shown (n = 3). *B*, H1299 cells were treated with or without Aurora A kinase inhibitor, MLN8237 (100 nM). Seahorse assays were performed to evaluate the glycolytic flux. ECAR over time (left panel) and ECAR in different stages of the measurement (right panel) were shown (n = 3). *C*, the glycolytic tracing assay was performed with ^13^C-labeled glucose in cells used in (*A*) (left panel). The relative abundance of the ^13^C-labeled glycolytic metabolites: G6P, PEP, serine, and IMP were shown (right panel). *D*, the glycolytic tracing assay was performed with ^13^C-labeled glucose in cells with or without Aurora A overexpression. The relative abundance of serine was shown. *E*, Flag-Aurora A was coexpressed with HA-PKM2 in H1299 cells. Extracts of H1299 cells with or without the treatment of serine/glycine starvation for 24 h were incubated with Flag beads. The elutes were immunoblotted with the indicated antibodies (left panel). Quantified data were shown (right panel) (n = 3). *F*, proximity ligation assay (PLA) was performed in H1299 cells with Aurora A and PKM2 antibodies upon the treatment of serine/glycine starvation for 24 h. DAPI and PCNA were cell cycle indicators (left panel). The scale bar represents 10 μm. The PLA signals per cell were quantified (right panel) (0 h, n = 29; 24 h, n = 27). DAPI, 4′,6-diamidino-2-phenylindole; ECAR, extracellular acidification; G6P, glucose 6-phosphate; IMP, inosine monophosphate; PEP, phosphoenolpyruvate.

Given the significant change of enzymatic activity of PKM2 upon phosphorylation at T45, we examined the metabolic function of phosphorylated PKM2. Seahorse assay showed that expression of PKM2 T45A and K433E mutants in H1299 decreased the glycolytic rate compared to PKM2 WT (Fig. 4*A*). Similarly, Aurora A inhibition and knockdown significantly reduced the glycolytic rate (Figs. 4*B* and S1*D*). In addition, glucose uptake and lactate secretion were found to be attenuated in PKM2 T45A expressing cells (Fig. S1*E*), while being promoted by Aurora A overexpression in biochemical assays (Fig. S1*F*).

As glycolytic intermediates also provide building blocks for biosynthesis in cancer cells (1), we investigated whether levels of these metabolites changed with the expression of PKM2 T45A mutant. Quantification of the glycolytic metabolites showed that the levels of glucose 6-phosphate (G6P), 2-phosphoglycerate (2PG), and NADPH decreased upon the expression of PKM2 T45A mutant in H1299 (Fig. S1*E*). In line with the enzymatic activity data, the level of pyruvate increased in cells expressing the T45A mutant.

It is known that tumor cells often experience metabolic stress during proliferation (27, 28). To overcome such stress, c-*myc*, an important upstream transcription factor of Aurora A, reprograms various metabolic pathways (29, 30). Indeed, when H1299 lung cancer cells were cultured in serine/glycine–deprived medium, the expression of Aurora A markedly increased (Fig. S1*G*). MS data showed that the PKM2 T45A mutant significantly reduced the fraction of ^13^C-glucose-derived carbon into G6P, PEP, serine, and inosine monophosphate, while the level of external glucose increased under the condition of serine/glycine starvation (Fig. 4*C*). The level of serine from ^13^C-glucose-derived carbon also increased upon Aurora A overexpression (Fig. 4*D*). Interestingly, upon serine/glycine starvation, the interaction between PKM2 and Aurora A was significantly enhanced (Fig. 4, *E* and *F*). Together, these results demonstrated that Aurora A–mediated phosphorylation of PKM2 at T45 promotes glycolytic flux and the biosynthesis pathways.

### Phosphorylation of PKM2 at T45 is critical for Aurora A–mediated tumor proliferation

**Figure 5:**
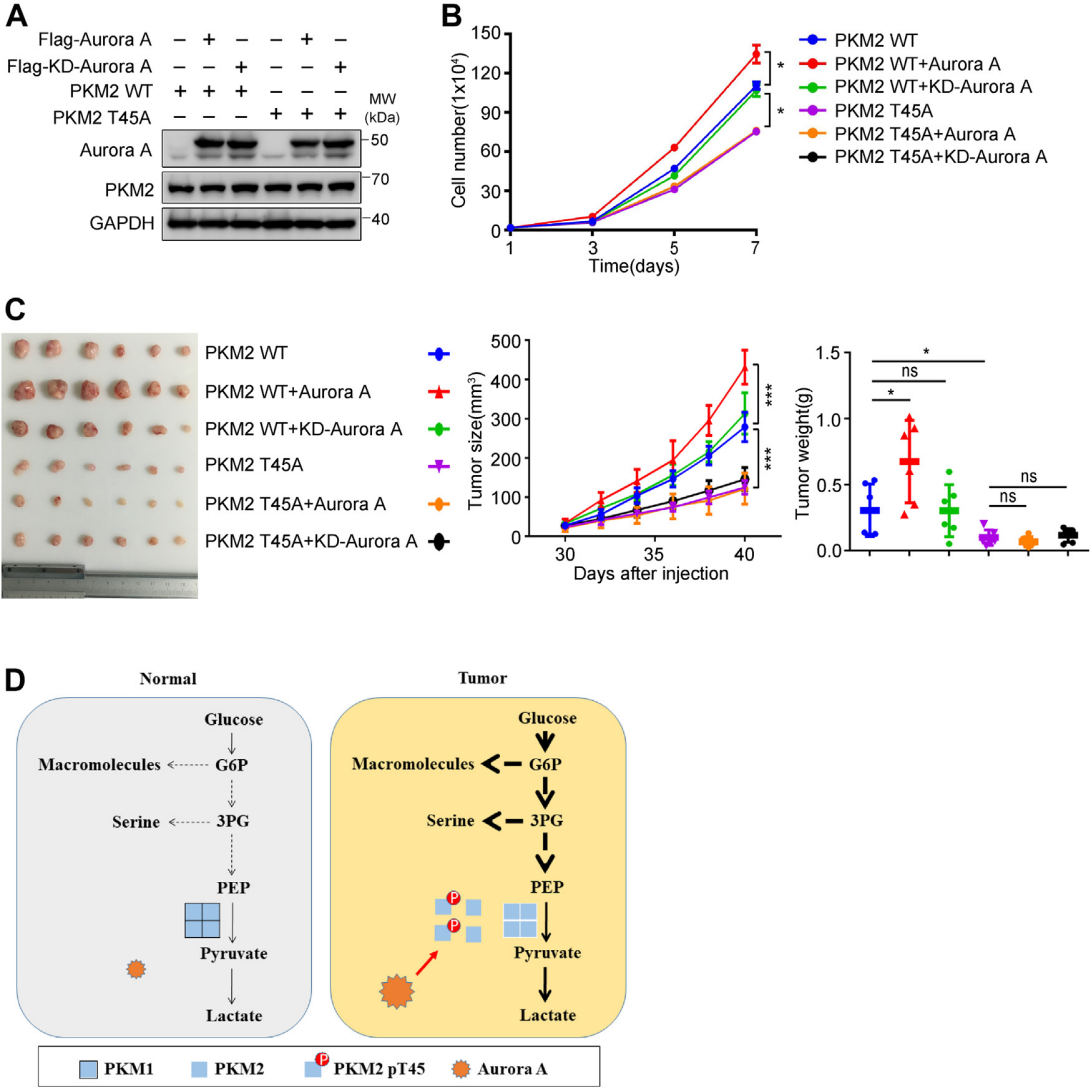
**Phosphorylation of PKM2 at T45 is critical for Aurora A–mediated tumor proliferation.***A*, Flag-Aurora A or kinase dead Aurora A (KD-Aurora A) were overexpressed in H1299 cells that had endogenous PKM2 knocked down and expressed shRNA-resistant Flag PKM2 WT or Flag-PKM2 T45A. The extracts of these cells were immunoblotted with the indicated antibodies. *B*, cell proliferation assay was conducted with the H1299 cells tested in (*A*) (n = 2). *C*, H1299 cells tested in (*A*) were inoculated in nude mice. The tumors at the endpoint (left panel), the growth curve of the xenograft (middle panel), and the weight of tumors (right panel) were shown (n = 6). *D*, a working model summarizes the function of Aurora A-PKM2 pathway in the regulation of Warburg effect in cancer cells.

To examine whether phosphorylation of PKM2 at T45 is critical for Aurora A–mediated tumor growth, we performed *in vitro* and *in vivo* experiments with H1299 cells that expressed either WT or kinase-dead (KD) Aurora A (Fig. 5*A*). In line with data from previous studies (25, 26, 31), stable overexpression of WT, but not KD Aurora A, increased cell proliferation *in vitro* (Fig. 5*B*). Moreover, expression of the PKM2 T45A mutant decreased cell proliferation, and overexpression of neither the WT nor KD Aurora A rescued the decrease of cell growth in the T45A mutant–expressing H1299 cells (Fig. 5*B*), illustrating that phosphorylation of PKM2 at T45 contributed to the protumor function of Aurora A. Similar results on tumor growth were also obtained in a H1299 xenograft mouse model (Fig. 5*C*). Overall, our data indicated that Aurora A–mediated phosphorylation of PKM2 at T45 is critical for tumor growth both *in vitro* and *in vivo*.

## Discussion

Compared to normal epithelial cells, cancer cells depend more on anabolic metabolism (1, 2). Therefore, during the cell cycle progression of cancer cells, cell cycle regulators have to efficiently facilitate the biosynthesis pathways. In this study, we uncovered a new interphase role of the mitotic kinase, Aurora A, in promoting anabolic metabolism of the glycolytic branching pathways by modulating the enzymatic activity of PKM2 during S phase. We demonstrated that Aurora A–mediated phosphorylation of PKM2 at T45 inhibits the enzyme activity of PKM2 through reducing its tetramerization, which not only increased the rate of glycolysis but also shifted glycolytic intermediates to act as building blocks for biosynthesis pathways, such as pentose phosphate pathway and the serine synthesis pathway (Fig. 5*D*). As Aurora A–mediated phosphorylation of PKM2 at T45 plays a critical role in glycolytic modulation, biomass synthesis pathways, and tumor progression, blocking phosphorylation of PKM2 at T45 by Aurora A inhibitor could be a promising therapeutic strategy for lung cancers.

It was reported that Aurora B can directly bind and phosphorylate PKM2 at T45 in U87 cells, which is required for its localization and interaction with MLC2 in the contractile ring region during cytokinesis (19). EGFR, RAS, and B-Raf mutants all enhance this PKM2 function that is critical for successful cell divisions and brain tumor progression. Intriguingly, we found the other Aurora kinase, Aurora A, also phosphorylates PKM2 at the same site in lung cancer cells. While Aurora B phosphorylates PKM2 during mitosis, the interaction between Aurora A and PKM2 occurs in the interphase and directly modulates the catalytic activity of PKM2 in glycolysis. Notably, expression of the PKM2 T45A mutant in lung cancer cells did not cause any defects during cytokinesis (Fig. S1*H*). The differences in PKM2 regulation by Aurora A and Aurora B may be due to differential upstream signaling and are context dependent. Aurora A could be upregulated upon nutrient shortage and promotes the shifting of glycolytic intermediates into biosynthesis pathways by controlling the last step of glycolysis in lung cancer. By contrast, in brain tumor, the hyperactive EGFR and its downstream signals activate Aurora B and PKM2 to facilitate cytokinesis. These results revealed variable interactions between Aurora kinases and PKM2 during different cell cycle phases and in different cancer cell types, pointing to context-specific temporal and spatial regulations of key cell cycle kinases and glycolytic enzymes.

In mammals, the PKM gene encodes PKM1 and PKM2 through alternative splicing of mutually exclusive exons that are identical in length but encode a 56 aa region that differs at 22 residues (32). PKM1 is expressed in most adult tissues and PKM2 is expressed during embryogenesis, regeneration, and the proliferation of most cancers (33). Among the 22 different amino acids, lysine 433 (K433) is a highly conserved residue and is present only in PKM2. As K433 of PKM2 is critical for the interaction with both Aurora A and Aurora B, both of the Aurora kinases likely only regulate PKM2, but not PKM1, in cancer cells.

Tumor cells often experience severe metabolic stress during proliferation (27). Here, we found that the expression of Aurora A kinase increased and the interaction between Aurora A and PKM2 was also significantly enhanced upon serine/glycine starvation. It was reported that serine/glycine starvation activates c-*myc* signaling, mRORC1, and serine synthesis (29, 30, 34), which could contribute to the transcriptional or posttranscriptional upregulation of Aurora A kinase (30) and its involvement in the redirection of glycolytic metabolites to biosynthesis. However, the precise mechanisms that connect metabolic stress and interaction of Aurora A and PKM2 remain unclear and require further study.

## Experimental procedures

### Cell culture

293T cells were cultured in Dulbecco's modified Eagle's medium (DMEM) (Gibco) supplemented with 10% fetal bovine serum (Gibco) and penicillin/streptomycin (100 IU/ml and 100 mg/ml, respectively; Beyotime Biotechnology). H1299 cells were cultured in RPMI1640 with the same supplements as DMEM. Cells were maintained at 37 °C in a 5% CO_2_ incubator and seeded into cell culture dishes 24 to 48 h before experimentation. For serine/glycine starvation experiments, cells were fed with DMEM deprived of the corresponding nutrients (29). Plasmid transfections were done with Lipofectamine 3000 (Invitrogen, #L3000015) or PEI (Polysciences, #23966). MLN8237 was from Selleck (S1133) and was dissolved in dimethyl sulfoxide at 1 mM.

### Antibodies

#### Primary antibodies

Rabbit-anti-PKM2(CST, 4053S, 1:2500), mouse anti-PKM2 (ProteinTech, 60268-1-Ig, 1:5000), rabbit anti-Aurora A (CST, #4718, 1:2000), mouse-anti-Aurora A (BD transduction, BD610939, 1:2000), rabbit-anti-p-Aurora A T288 (CST, #3079, 1:2000), mouse anti-FLAG (Transgene, #HT201, 1:2000), mouse anti-GST (ProteinTech, 66001-2-Ig, 1:5000), mouse-anti-HA (ProteinTech, 66006-I-Ig, 1:5000), mouse-anti-His(Transgene, #HT501, 1:5000), mouse anti-Aurora B (AIM, BD transduction, #611082, 1:2000), mouse anti-GAPDH (ProteinTech, 60004-I-Ig, 1:5000). Coralite488-conjugated PCNA (ProteinTech, CL488-10205, 1:100).

#### Secondary antibodies

Goat anti-rabbit or mouse horseradish peroxidase (Jackson Immuno-Research, 1:5000).

### Plasmids and recombinant proteins expression

Flag-Aurora A/B was constructed by cloning full-length Aurora A/B into the BglII/KpnI sites of p3×Flag-Myc-CMV-24 (Sigma–Aldrich). GST-Aurora A was constructed by cloning the full-length Aurora A into the BamH/XhoI sites of p-GEX-5X-3 (Sigma–Aldrich). HA-Aurora A was constructed by cloning the full-length Aurora A into the BamHI/EcoRI sites of pKH3 (Addgene). His-PKM2 was constructed by cloning the full-length PKM2 into the EcoRI/BamHI sites of pET-22b (Addgene). Flag-PKM2 was constructed by cloning the full-length PKM2 into the EcoRI/BamHI sites of p3×Flag-Myc-CMV-24 (Sigma–Aldrich). HA-PKM2 was constructed by cloning the full-length PKM2 into the EcoRI/BamHI sites of pKH3 (Addgene). A freshly transformed colony was used to initiate a small volume of liquid culture in LB medium with 100 μg/ml ampicillin. This culture was used to inoculate a large volume of the same medium and grown until an absorbance at 600 nm of 0.5 was reached. Protein expression was induced by adding 0.5 mM IPTG and bacteria continued to grow with shaking at 16 °C for 20 h. Afterward, bacteria were lysed and sonicated, followed by centrifugation. The supernatant was stored with 15% glycerol at −80 °C.

### PKM2 knockdown and reintroduction

pGIPZ human PKM2 shRNA was generated with CATCTACCACTTGCAATTA oligonucleotide that targets the exon 10 of the PKM2 transcript. Flag-tagged human PKM2 WT and PKM2 T45A mutant containing two silent nucleotide substitutions in the sequence corresponding to the shRNA-targeted region were cloned into the pGIPZ system and cotransfected into 293T cells together with vectors expressing the gag and vsvg genes. Retroviral supernatant was harvested 48 h after initial plasmid transfection and mixed with polybrene (8 mg/ml) to increase the infection efficiency. H1299 cells were infected with retrovirus and selected in puromycin (2 μg/ml) for 1 week (35).

### *In vitro* kinase reactions, autoradiography, and mass spectrometry

For *in vitro* kinase assay, the reaction was performed in 20 μl of 1 × kinase buffer (50 mM NaCl, 2 mM EGTA, 25 mM Hepes, pH7.2, 5 mM MgSO_4_, 1 mM DTT) containing 100 ng of human recombinant Aurora A (Life Technologies Corporation, #PV3612), 1 μg of His-tagged protein, and 50 μM ATP. After incubation for 30 min at 30 °C, the reaction was stopped with 5 × loading buffer. The protein mixture was then heated at 95 °C for 5 min and separated by 10% SDS-PAGE. The bands containing His-PKM2 were cut and analyzed by MS (Shanghai Applied Protein Technology Co Ltd). For the ^32^P assay, an additional 5 μCi of γ-^32^P ATP was added into the kinase reaction mixture. After *in vitro* kinase reaction, the reaction mixture was heated at 95 °C for 5 min and separated by 10% SDS-PAGE. For the autoradiography assay, the gels were dried, followed by exposure to X-ray film.

### Immunoprecipitation, GST pull-down assays, and immunoblotting

For co-IP assay, 293T cells cotransfected with Flag-tagged and HA-tagged protein were lysed in immunoprecipitation buffer (50 mM Tris–HCl, pH7.4, with 250 mM NaCl, 1 mM EDTA, 50 mM NaF, and 0.5% Triton X-100, together with protease inhibitors). The Flag-tagged proteins were precipitated with anti-Flag M2 resin following the manufacturer’s instructions (Sigma, M8823). For GST pull-down assays, *Escherichia coli* were lysed in lysis buffer (10 mM Tris pH7.4, 150 mM NaCl, 0.25% NP-40, 10 mM EDTA, 5 mM DTT, 5 mM PMSF, and protease inhibitors) by sonication. GST-tagged proteins precipitated by affinity column chromatography were carried out using amylose resin following the manufacturer’s instructions (Transgene, #DP201-01). His-tagged proteins precipitated by affinity column chromatography were carried out using nickel column following the manufacturer’s instructions (Qiagen, 30210). Purified His-PKM2 protein was incubated with amylose resin containing GST-tagged protein for 2 h at 4 °C and eluted with 10 mM GSH in 50 mM Tris (pH = 8.0). For immunoblotting, proteins were resolved by SDS-PAGE and transferred to polyvinylidene difluoride membranes (Millipore). Immunoblots were developed using Western Lightning Chemiluminescence Reagent Plus (Advansta) and exposed with chemiluminescence machine (36).

### DSS crosslinking assay

DSS crosslinking was performed according to published protocol. After washing with cold 1 × PBS, cells were suspended with conjugation buffer (20 mM Hepes, pH = 8.0). DSS solution in dimethyl sulfoxide was added to the cell suspension to a final concentration of 1.5 mM. After incubating at room temperature (RT) for 30 min, the samples were boiled and used for Western blotting (37).

### Pyruvate kinase (PK) activity assay

The PK activity was assessed using a PK assay method (Biovision, K709) according to the manufacturer’s protocol. All values were normalized on the basis of the Bradford protein assay.

### Glucose uptake and lactate secretion assay

Cells were seeded onto 96-well plates or 6-well plates, and after 6 h, the culture medium was replaced with fresh complete medium and incubated for additional 48 h. The media were then collected for measurement of glucose and lactate concentration and cells were harvested for protein lysates. Glucose levels were determined using a glucose assay kit (Biovision, K676). Lactate levels were determined using a lactate assay kit (Biovision, K607), according to the manufacturer’s instruction. All values were normalized on the basis of the Bradford protein assay.

### The extracellular acidification (ECAR) measurement

Measurements of ECAR in H1299 cells were performed with the Seahorse XF96e analyzer (Seahorse Bioscience), according to the manufacturer’s instruction. Briefly, 6000 cells were seeded per well overnight in a 96-well XF cell culture microplate in growth medium. ECAR was measured with an XF96 analyzer in XF base medium containing 4 mM glutamine (pH = 7.35) following sequential additions of glucose (10 mM), oligomycin (1 mM), and 2-DG (50 mM). Data were analyzed by a Seahorse XF Glycolysis Stress Test Report Generator.

### Quantification of glycolytic intermediates

Levels of pyruvate, G6P, and 2PG were analyzed using Pyruvate (Bioassay, #EPYR), G6P (Biovision, #K657), and 2PG (Biovision, #K778) Assay Kit, respectively, according to the manufacturer’s protocol. All values were normalized on the basis of the Bradford protein assay.

### GC-MS analysis of metabolites

Cells were incubated in the culture medium supplemented with 5 mM ^13^C_6_-glucose (Sigma–Aldrich, 389374) under the indicated conditions. Metabolites were extracted from cells as previously described (38). Briefly, each group of cells was collected and immediately flash frozen in liquid N_2_, metabolites were extracted with ice-cold methanol, and lysates were centrifuged at 18,000*g* for 15 min to remove protein. The supernatant was dried in an evaporator and resuspended in 200 μl pyridine. Metabolites were further derivatized by addition of 50 μl MTBSTFA containing 1% t-BDMCS at 60 °C for 1 h. Samples were analyzed using Agilent 5MS column in the Agilent 7890/5975C GC/MS system (Agilent Technologies). Peaks representing each metabolite were extracted and integrated using MassHunter software (Agilent Technologies). ^13^C-labeled metabolite data were presented as percentage of ^13^C-labeled metabolites, which was calculated by dividing the labeled ions with total ion intensity. The labels were irrespective of carbon position. The distribution of mass isotopologs was corrected for natural abundance. We used IsoCor, a scientific software designed for the purpose of isotope labeling experiments, to correct the raw MS data for both all naturally occurring isotopes and purity of the isotopic tracer. The website for the software (IsoCor) is http://metasys.insa-toulouse.fr/software/isocor/.

### Nano LC-MS/MS

The *in vitro* kinase assay and FLAG immunoprecipitation were conducted as described previously. The samples were reduced with 10 mM DTT in 50 mM ammonium bicarbonate at 55 °C for 30 min and then alkylated with 30 mM of iodoacetamide for 30 min in dark. After the aforementioned process, 2 μg of trypsin (Promega, V511A) was added to sample for overnight digestion at 37 °C. After digestion, the peptide sample was desalted and analyzed with Orbitrap Exploris 480 mass spectrometer equipped with Easy-nanoLC 1200. The raw file was analyzed with pFind Studio 3. The human database was from UniProt (Proteome ID: UP000005640). Phosphorylation (S/T, +79.9663Da) and oxidation (M, +15.9949Da) modification were included as variable modification. Carbamidomethyl (C, +57.0215Da) was set as fix modification.

### Cell proliferation assays

For proliferation assay, 1 × 10^4^ cells were seeded in triplicate in 12-well plates and cell numbers were counted every 24 h over a 7 day period.

### PLA

Cells were seeded in the μ-slide angiogenesis glass bottom (Ibidi, #81507). The next day, the cells were treated with or without serine/glycine starvation for 24 h, washed with PBS, and fixed with 4% paraformaldehyde in PBS at RT for 20 min. The remaining steps were performed according to published protocol (39). Images were acquired with a Delta Vision microscope (GE Healthcare) equipped a 60 × objective lens, numerical aperture = 1.42.

### Structure analysis and molecular dynamics simulation

The complex structure of PKM2 and serine (Protei Data Bank entry 4B2D) was used to set up molecular dynamics simulations. The first one is for the WT structure. In the second simulation, T45 was phosphorylated (pT45). Molecular dynamics simulations were performed as described previously (25).

### SEC coupled with static laser light scattering (SEC-multiangle light scattering)

The WT and T45A mutant of PKM2 protein were concentrated to 1 mg/ml in the working buffer (150 mM NaCl, 20 mM Tris, pH = 7.5) and verified by SEC-multiangle light scattering. The experiments were performed on a multiangle light scattering detector (Wyatt) connected in line with Superdex 200 Increase column (GE Healthcare) run by ÄKTA pure 25 (GE Healthcare) at a flowrate of 0.3 ml/min. The Astra software (Version 7.0.1.24 https://www.wyatt.com/products/software/astra.html) was used to collect and analyze data.

### Xenograft

All animal studies were conducted with approval from the Animal Research Ethics Committee of the University of Science and Technology of China. For xenograft experiments, 5 × 10^6^ H1299 cells stably expressing shRNA-resistant PKM2 WT and T45A were injected subcutaneously into 5-week-old male nude mice (SJA Laboratory Animal Company). The tumor volumes were measured using digital calipers every 3 days and calculated using the equation: length (mm) × width (mm) × depth (mm) × 0.52.

### Bioinformatic analysis

The survival analyses were performed using the GEPIA2 bioinformatics website (http://gepia2.cancer-pku.cn/#analysis) classified by median expression level.

### Statistical analysis

All the data statistics are presented as mean ± SD. The significance of differences was determined using Student’s *t* test. *p* < 0.05 was considered to be statistically significant.

## Data availability

The mass spectrometry proteomics data (Figs. 2*C* and S1*B*) have been deposited to the ProteomeXchange Consortium *via* the PRIDE partner repository with the dataset identifier PXD036575. The supporting information data underlying Figs. 1*C*, 2*D*, 2*E*, 3,*A*–*F*, 4,*A*–*F*, 5*B*, 5*C*, S1, *C*–*F* and S1*H* are provided as source Data file. The uncropped blot for Figs. 1, *A* and *B*, 2A, 2,*C*–*E*, 3*E*, 4*E*, 5*A* and S1*G* are shown in the supporting information (Fig. S2).

## Supporting information

This article contains supporting information.

## Conflict of interest

The authors declare that they have no conflicts of interest with the contents of this article.

## Supporting information

Supporting Information

Supplemental Fig. S1 Aurora A-mediated PKM2 phosphorylation promotes tumor growth**.** A. Alignment of amino acid sequences containing threonine 45 of PKM2 from several model species: *Homo Sapiens*, *Pan troglodytes*, *Rhesus Macaque*, *Lupus familis*, *Mus musculus*, *Rattus norvegicus*, *Brachydanio rerio* and *Takifugu rubipres*. B. In vitro kinase assay was performed with recombinant His-PKM2 and GST-Aurora A or His-PKM2 and GST-KD-Aurora A (kinase dead Aurora A, D274A). After reaction, the protein was subjected to MS. The number of PSMs (the peptide-spectrum matches) containing PKM2 T45 or PKM2 pT45 were counted. C. The activities of PK in extracts of H1299 cells with Aurora A overexpression or MLN8237 (100 nM) treatment were measured (n = 2). D. Aurora A was knocked down in H1299 cells. Seahorse assays were performed to evaluate the glycolytic flux. ECAR over time (left panel) and ECAR in different stages of the measurement (right panel) were shown (n = 2). E. The levels of several glycolytic intermediate metabolites, including glucose-6-phosphate (G6P) (n = 4), 2-phosphoglycerate (2PG) (n = 5), pyruvate (n = 4) and NADPH (n = 3) were determined in H1299 cells used in Figure 4A. F. The relative glucose uptake (left panel) (n = 5) and lactate production (right panel) (n = 4) were measured in H1299 cells with or without Aurora A overexpression. G. Extracts of H1299 cells with or without the treatment of serine/glycine starvation for 24 hours were immunoblotted with the indicated antibodies. H. Time-lapse microscopy of H1299 cells that had endogenous PKM2 knocked down and expressed shRNA-resistant Flag-PKM2 WT or Flag-PKM2 T45A (left panel). Quantified data were shown (right panel) (n = 3). Scale bar, 10 μm.
