# Supplementary figures and images for: Aurora A–mediated pyruvate kinase M2 phosphorylation promotes biosynthesis with glycolytic metabolites and tumor cell cycle progression
Source: J Biol Chem. 2022 Oct 2;298(11):102561. doi: 10.1016/j.jbc.2022.102561 (PMC9637814; doi:10.1016/j.jbc.2022.102561)

Figure S1

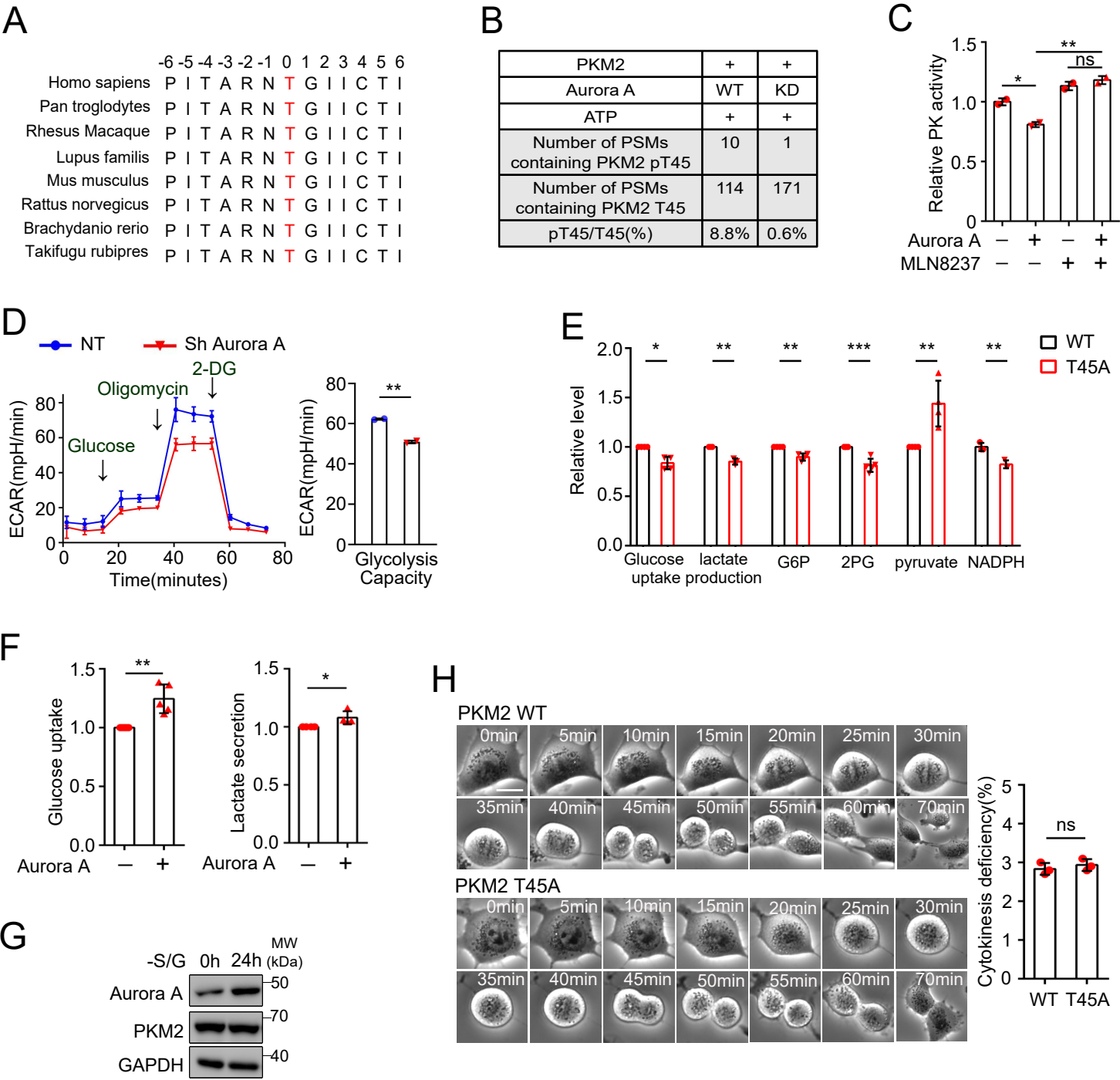

Supplement: Supplemental Fig. S1 — Aurora A-mediated PKM2 phosphorylation promotes tumor growth. A. Alignment of amino acid sequences containing threonine 45 of PKM2 from several model species: Homo Sapiens, Pan troglodytes, Rhesus Macaque, Lupus familis, Mus musculus, Rattus norvegicus, Brachydanio rerio and Takifugu rubipres. B. In vitro kinase assay was performed with recombinant His-PKM2 and GST-Aurora A or His-PKM2 and GST-KD-Aurora A (kinase dead Aurora A, D274A). After reaction, the protein was subjected to MS. The number of PSMs (the peptide-spectrum matches) containing PKM2 T45 or PKM2 pT45 were counted. C. The activities of PK in extracts of H1299 cells with Aurora A overexpression or MLN8237 (100 nM) treatment were measured (n = 2). D. Aurora A was knocked down in H1299 cells. Seahorse assays were performed to evaluate the glycolytic flux. ECAR over time (left panel) and ECAR in different stages of the measurement (right panel) were shown (n = 2). E. The levels of several glycolytic intermediate metabolites, including glucose-6-phosphate (G6P) (n = 4), 2-phosphoglycerate (2PG) (n = 5), pyruvate (n = 4) and NADPH (n = 3) were determined in H1299 cells used in Figure 4A. F. The relative glucose uptake (left panel) (n = 5) and lactate production (right panel) (n = 4) were measured in H1299 cells with or without Aurora A overexpression. G. Extracts of H1299 cells with or without the treatment of serine/glycine starvation for 24 hours were immunoblotted with the indicated antibodies. H. Time-lapse microscopy of H1299 cells that had endogenous PKM2 knocked down and expressed shRNA-resistant Flag-PKM2 WT or Flag-PKM2 T45A (left panel). Quantified data were shown (right panel) (n = 3). Scale bar, 10 μm. [file mmc2.pdf]
