# Supplementary material for: Aurora A–mediated pyruvate kinase M2 phosphorylation promotes biosynthesis with glycolytic metabolites and tumor cell cycle progression
Source: J Biol Chem. 2022 Oct 2;298(11):102561. doi: 10.1016/j.jbc.2022.102561 (PMC9637814; doi:10.1016/j.jbc.2022.102561)
Supplement: Supplemental Fig. S2 [file mmc3.pdf]

Figure S2. Full western blots. Boxes in black indicate selected western blot results.

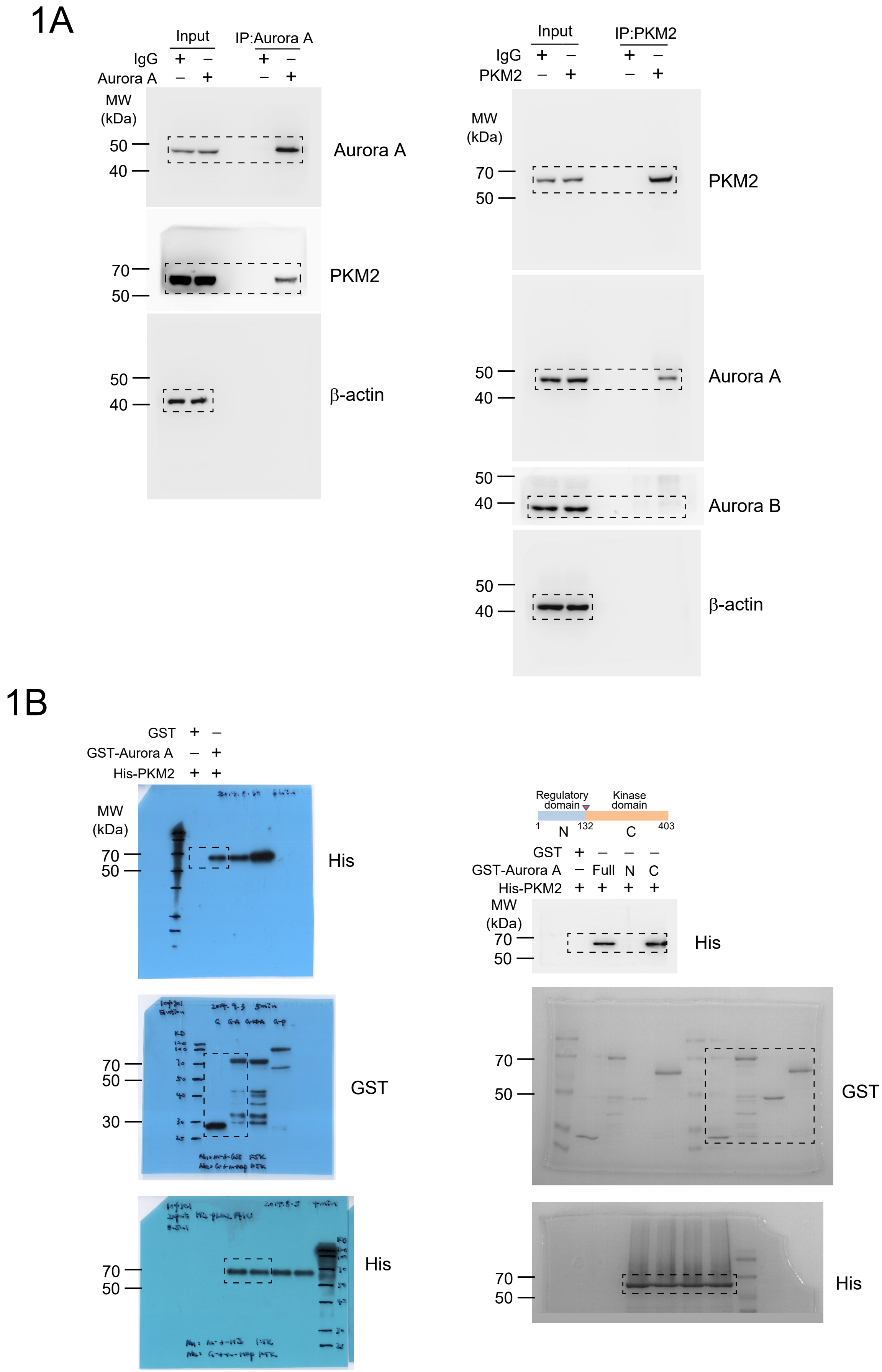

2A

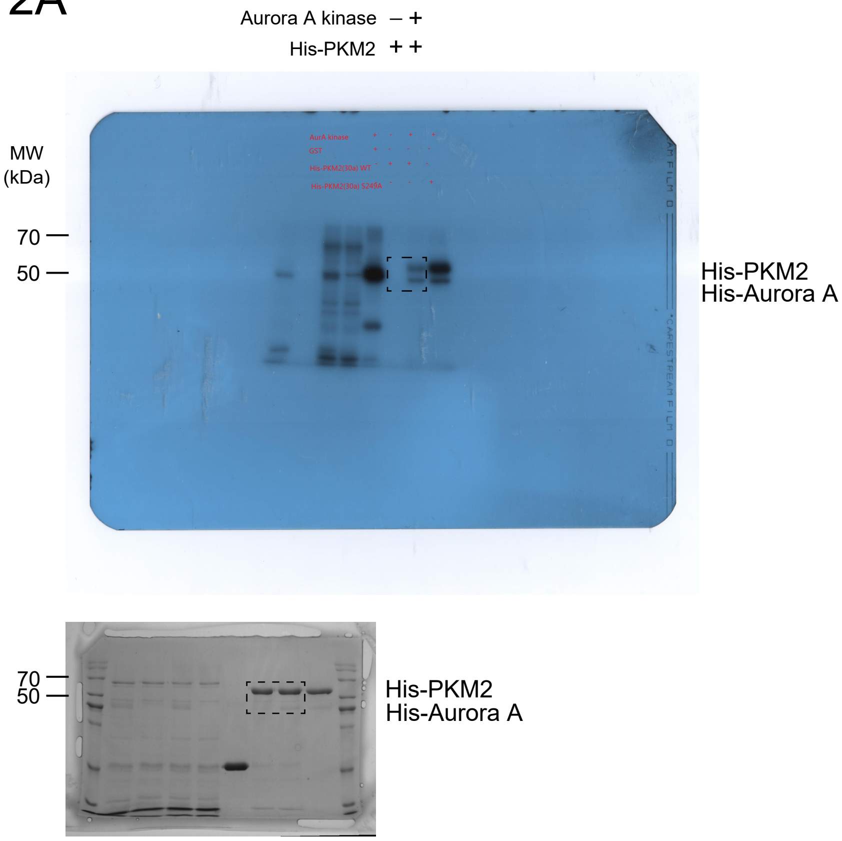
Aurora A kinase – +
His-PKM2 + +
MW
(kDa)
70
50
His-PKM2
His-Aurora A
70
50
His-PKM2
His-Aurora A

2C

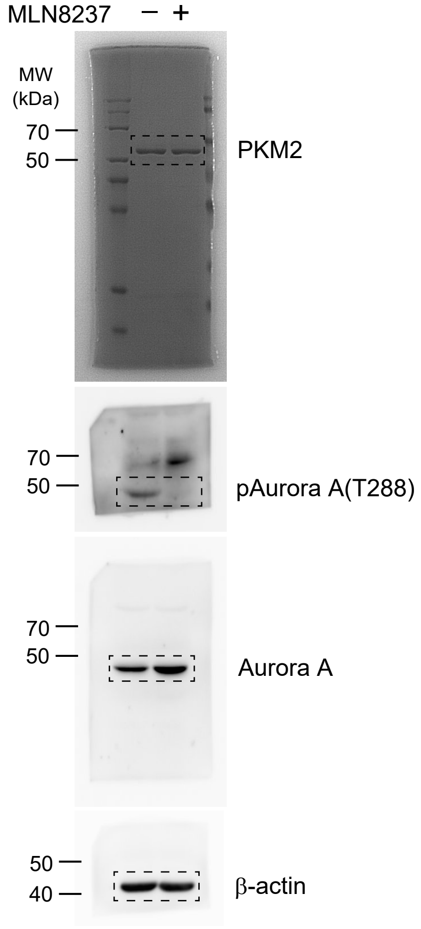
MLN8237 – +
MW
(kDa)
70
50
PKM2
70
50
pAurora A(T288)
70
50
Aurora A
50
40
β-actin

2D

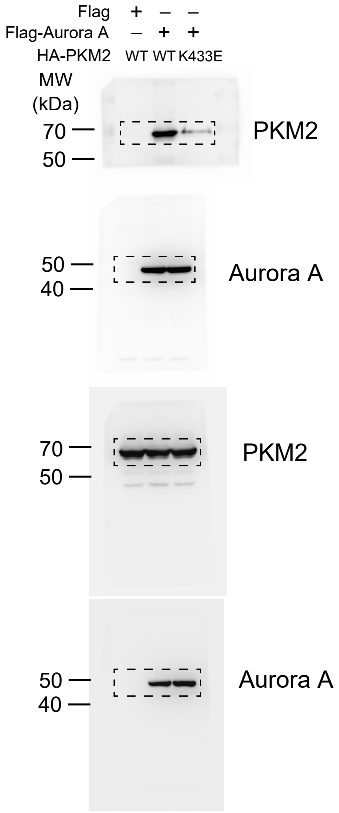
Flag + – –
Flag-Aurora A – + +
HA-PKM2 WT WT K433E
MW
(kDa)
70
50
PKM2
50
40
Aurora A
70
50
PKM2
50
40
Aurora A

2E

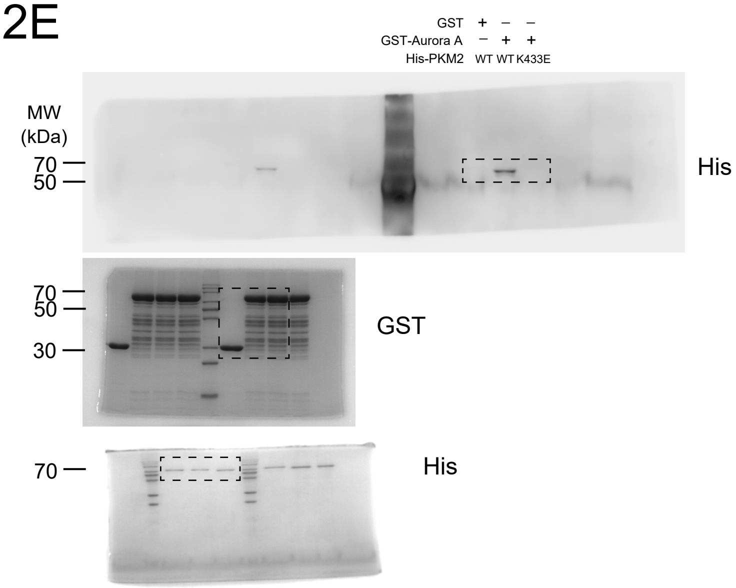
GST + – –
GST-Aurora A – + +
His-PKM2 WT WT K433E
MW
(kDa)
70
50
His
70
50
30
GST
70
His

3E

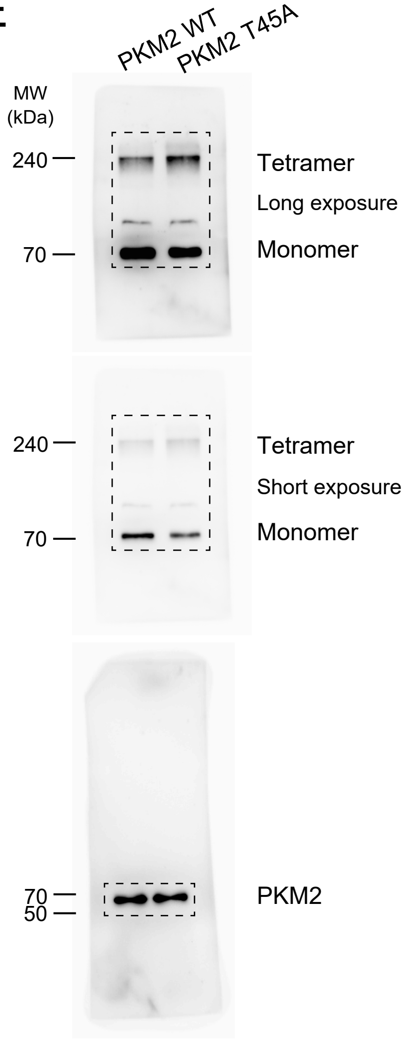
PKM2 WT
PKM2 T45A
MW (kDa)
240
70
Tetramer
Long exposure
Monomer
240
70
Tetramer
Short exposure
Monomer
70
50
PKM2

4E

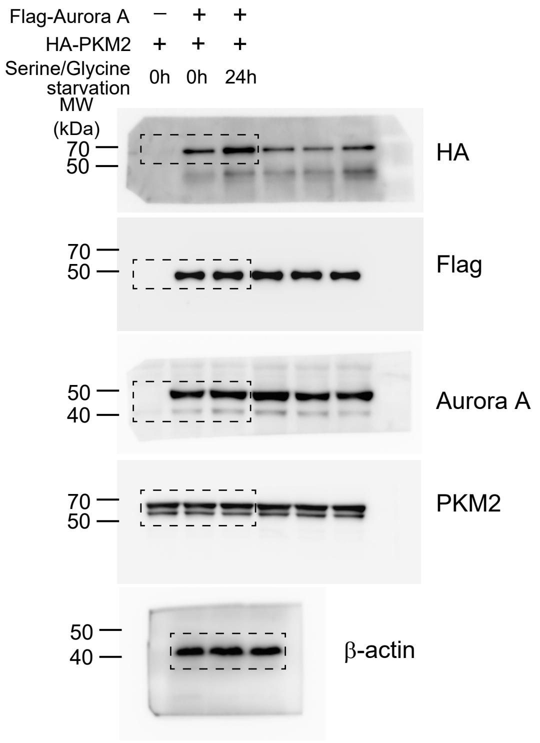
Flag-Aurora A – + +
HA-PKM2 + + +
Serine/Glycine starvation 0h 0h 24h
MW (kDa)
70
50
HA
70
50
Flag
50
40
Aurora A
70
50
PKM2
50
40
β-actin

5A

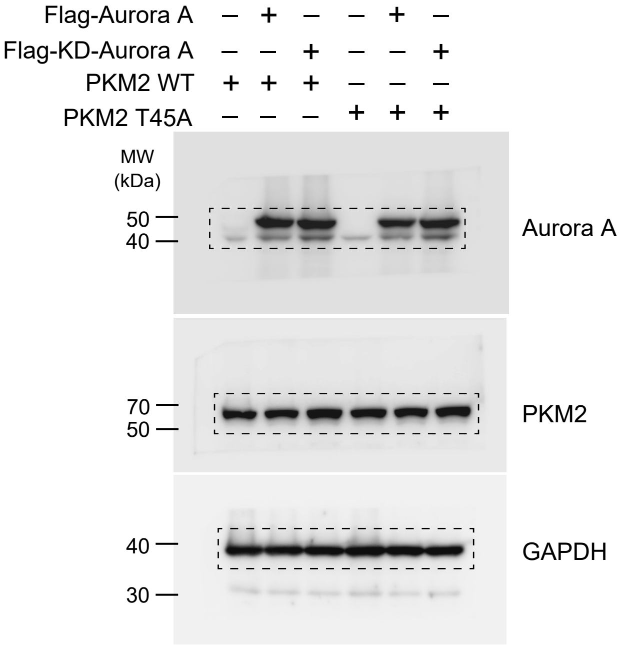
Flag-Aurora A – + – – + –
Flag-KD-Aurora A – – + – – +
PKM2 WT + + + – – –
PKM2 T45A – – – + + +
MW (kDa)
50
40
Aurora A
70
50
PKM2
40
30
GAPDH

S1G

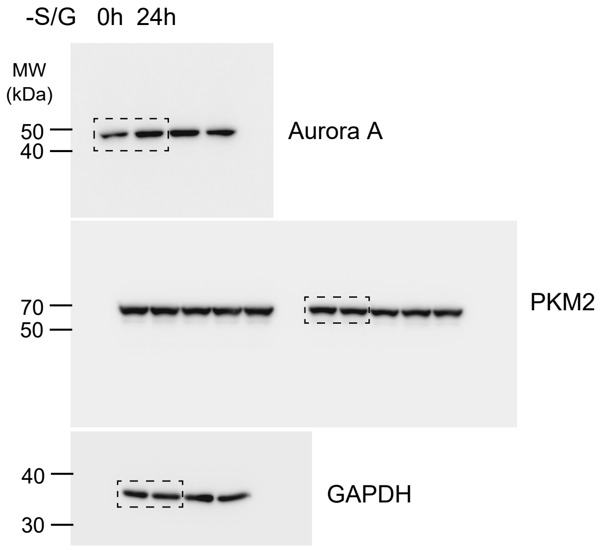
-S/G 0h 24h
MW (kDa)
50
40
Aurora A
70
50
PKM2
40
30
GAPDH
